# A 50-year-old with rapid neuropsychiatric deterioration and choreiform movements

**DOI:** 10.1136/practneurol-2016-001481

**Published:** 2017-01-24

**Authors:** Graham Andrew Mackay, Stewart Campbell, Ravi Jampana, Jonathan Cavanagh

**Affiliations:** 1 Department of Neurology, Queen Elizabeth University hospital, Glasgow, UK; 2 Department of Gastroenterology, Hairmyres hospital, East Kilbride, UK; 3 Department of Neuro-radiology, Queen Elizabeth University hospital, Glasgow, UK; 4 Mental Health and Wellbeing, Sackler Institute, Neurology block, Queen Elizabeth University hospital, Glasgow, UK

**Keywords:** Lupus vasculitis, Central Nervous System, Encephalitis, Chorea, Cognition

A 50-year-old man presented acutely to the hospital with behavioural disturbance, choreiform movements and profound nihilistic delusions. He reported recent drug and alcohol abuse, and also apparent involvement in several recent criminal activities, for which he felt he should be punished. He arrived alone at the hospital after a concerned neighbour had called an ambulance. His initial level of agitation prevented formal cognitive testing. However, he was alert, verbally responsive and could obey commands. He was afebrile with normal observations and normal plasma glucose. Although his examination was challenging, the only abnormal neurological findings were bilateral choreiform upper limb movements.

**Question 1**

What should you do now?

## Comments

Obtaining a collateral history is essential in establishing baseline function in people presenting with neuropsychiatric disturbance. This can help us focus on the differential diagnoses and plan for investigation. It can also avoid repeating previous investigations.

The admitting team contacted his brother, his next of kin. The patient was an unemployed ex-builder. One year earlier, he had consulted a movement disorder doctor with a high-frequency tremor and brief involuntary movements. Investigations at this time included a normal MR scan of the brain and routine blood tests, including thyroid function tests and serum calcium. In the 2 months before admission, his family had noticed occasional repetitive conversation. However, he still lived independently and attended training courses. His agitation and disorientation had developed over the 24 hours before presentation. There was no family history of dementia or of involuntary movements.

**Question 2**

What is the differential diagnosis?

**Question 3**

What initial investigations would you plan?

Causes of cognitive impairment and chorea (box 1).

Urine screening for illicit drugs was negative. The following were normal: blood film, renal function, serum calcium, thyroid function tests and serum C-reactive protein; serum antistreptolysin O titres were negative. Urinalysis was normal. MR scan of the brain, including diffusion-weighted and susceptibility sequences, showed only minor non-specific white matter abnormalities. Cerebral MR angiography was also normal.

Box 1 **Differential diagnosis for rapid cognitive decline and chorea**

- Drug-induced chorea, such as cocaine, amphetamines, lithium, methylphenidate, benzodiazepines, neuroleptics)
- Basal ganglia lesion
  - Stroke (sub-thalamic nucleus)
  - Space occupying lesion
- Postinfective, such as herpes simplex virus, Sydenham’s chorea, paediatric autoimmune neuropsychiatric disorders associated with streptococcal infections, AIDS
- Thyrotoxicosis
- Systemic lupus erythematosus/antiphospholipid syndrome
- Polycythaemia rubra vera
- Chorea gravidarum
- Autoimmune encephalitis (NMDAr)
- Variant Creutzfeldt-Jakob disease
- Inherited conditions
  - Huntington’s disease
  - Wilson’s disease
  - Neuroacanthocytosis, Macleod’s syndrome
  - Neuroferritinopathy/pantothenate kinase-associated neurodegeneration
  - Kufs disease, corticobasal degeneration, dentatorubral-pallidoluysian atrophy, Lesch-Nyhan syndrome
  - Friedreich’s ataxia
  - Mitochondrial disorders

## Comments

Before lumbar puncture, he was noted to have a prolonged activated partial thromboplastin time but normal prothrombin time. This did not correct with vitamin K, prompting his medical team to look for the lupus anticoagulant and for serum antinuclear antibodies. His lumbar puncture showed 20 × 10^6^red cells/l (0) and 10 × 10^6^ white cells/l (≤5) (lymphocytes) with normal cerebrospinal fluid (CSF) protein and glucose concentrations. Oligoclonal band testing was equivocal. His serum was positive for antinuclear antibody (1/640), anti-double-stranded DNA (ELISA) at 840.4 U/mL (<35) and positive anti-DNA immunophoresis. He had a prolonged activated partial thromboplastin time and positive testing for the lupus anticoagulant (Dilute Russell"s viper venom time (DRWT)). He had positive anticardiolipin IgG 16.9 U/mL (normal <10), but normal IgM levels. His serum complement C3 levels were initially low at 0.71 g/l (normal >0.8), with normal C4 levels.

**Question 4**

What is the most likely diagnosis?

**Question 5**

How would you initially treat him?

## Comments

We made the presumptive diagnosis of diffuse neuropsychiatric lupus. He was originally treated with 5 days of intravenous methylprednisolone, with clear initial improvement in his agitation and lessening of his involuntary movements. He scored 54/100 on the revised Addenbrooke's Cognitive Assessment (ACE-R). The rheumatology team started him on tapering oral corticosteroids, followed by intravenous cyclophosphamide 500 mg weekly for 6 weeks. He did not improve and, if anything, deteriorated further with more intrusive depressive thoughts, worsening cognitive function and a return of the chorea. He began complaining of visual hallucinations of a dog in his room.

**Question 6**

How would you manage him now?

**Question 7**

Are there any other treatment options and how might you monitor their impact?

## Comments

**Figure 1:**
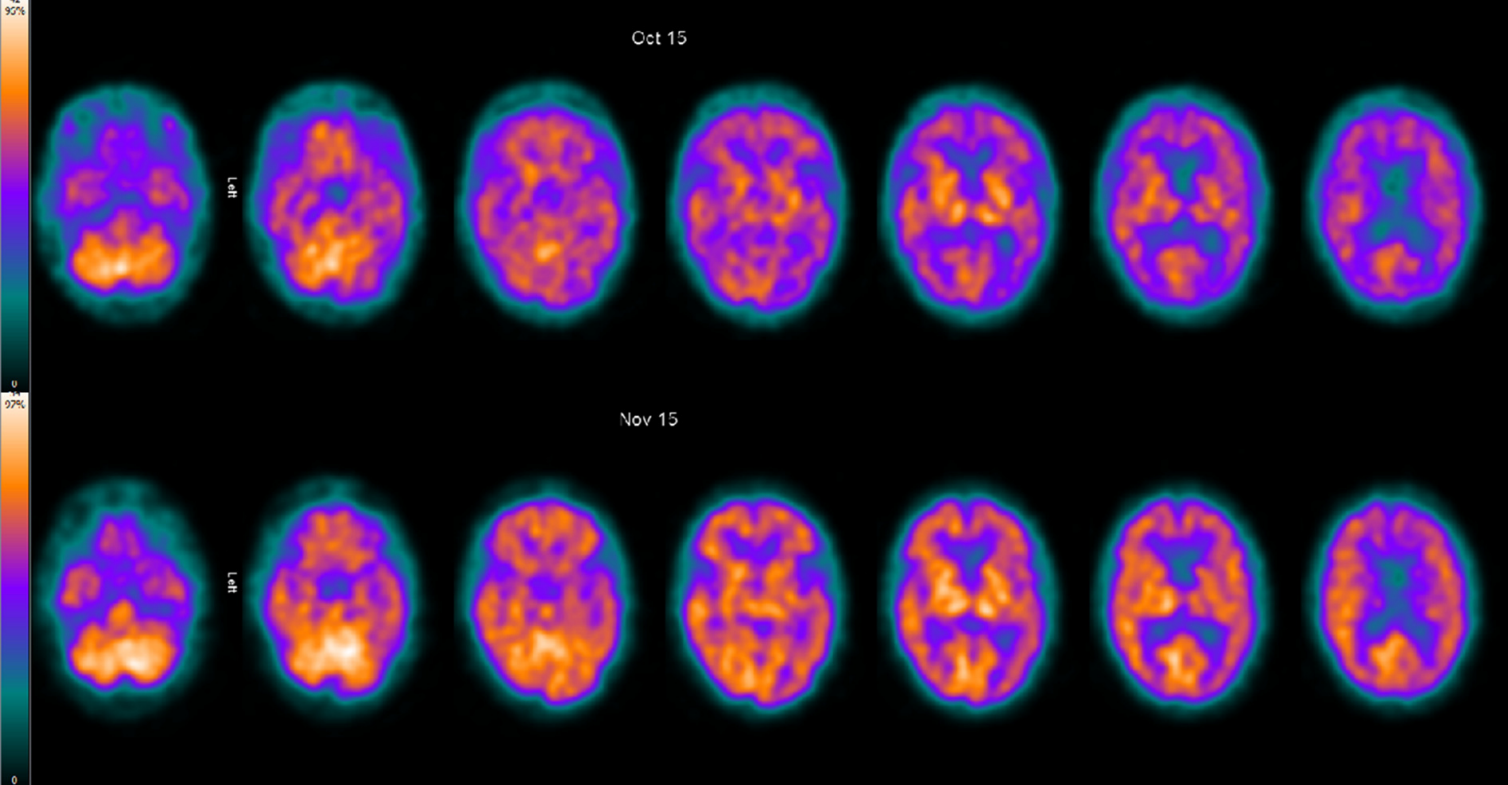
Cerebral perfusion single photon emission CT using intravenously injected ^Tc^99m HMPAO. Axial projections. Pretreatment (top row) and post-treatment (bottom row) scans. As shown in the colour scale to the left, the brighter yellow the colour the greater the perfusion. Cortical perfusion is generally reduced on the pretreatment scan, but significantly improved after treatment.

Given his emerging depressive psychotic symptoms, we arranged for psychiatric evaluation. He was treated with venlafaxine and then additionally quetiapine, with a gradual impact on his depressive symptoms and resolution of the hallucinations. At this stage, he had five plasma exchange treatments without clear effect. An hexamethylpropyleneamine oxime (HMPOA) single photon emission CT (SPECT) showed bilateral cortical hypoperfusion, most prominent over the left temporal lobe (figure 1). Given his static and severely impaired cognitive function, we started him on intravenous rituximab. He received four weekly doses of 600 mg, having been screened for relevant infections (HIV, hepatitis B and C, and tuberculosis). His cognitive function significantly improved, the ACE-R score rising to 80/100. Repeat HMPOA SPECT showed increased perfusion (figure 1). During this time, he also received low-molecular-weight heparin for 6 weeks, given the associated antiphospholipid antibodies although vascular imaging never showed evidence of thrombosis. He now takes hydroxychloroquine as maintenance immunosuppressive therapy along with the venlafaxine and quetiapine. He manages self-care, but 12 months after initial presentation he still cannot yet live independently, requiring some supervision. His depression has resolved and his personality has returned to normal.

## Discussion

Most neuropsychiatric lupus events occur at onset or within an year of onset (50%–60%).^1^ The American College of Rheumatology criteria for classifying neuropsychiatric lupus is very broad and includes 19 potential neurological and psychiatric syndromes (table 1), including both central and peripheral syndromes.^2^ The spectrum of events varies from headache, mood disorders and mild cognitive impairment to severe cases.^3^ Severe cognitive cases such as this comprise only 3%–9% of neuropsychiatric lupus.^1 4^ Chorea is the most common movement disorder and is associated with antiphospholipid antibodies, but occurs in less than 4% of cases.^1 3^ Severe neuropsychiatric cases have either focal or diffuse presentations.^5–7^ The focal cases have ischaemic, thrombotic or demyelinating lesions. Our patient had a diffuse presentation, similar to several previously described cases. However, these diffuse cases were variably described as having severe neuropsychiatric lupus or acute confusional state presentations, making it difficult to compare the reported cases.

These diffuse cases are most likely immune mediated, possibly with several autoantibodies, including antiribosomal-P, anti-DNA, antiphospholipid, NMDA NR2 and GABA-B antibodies, supporting this assertion.^8 9^

Patients with suspected neuropsychiatric lupus should have an MR scan of brain^10^ to exclude several differential diagnoses, including demyelination, ischaemia or thrombosis. Over half of neuropsychiatric lupus cases have normal MR brain scans.^5 8^ Non-specific white matter abnormalities are common in systemic lupus erythematosus and in neuropsychiatric lupus cases, and are non-discriminatory.^5 11^ Serial MR brain scans in diffuse neuropsychiatric lupus cases may show rapidly progressive generalised brain atrophy.^12^ Lumbar puncture is important to exclude central nervous system (CNS) infection; it can show an inflammatory response but can be normal. Some patients have positive CSF oligoclonal bands and other CSF immune biomarkers, such as anti-DNA antibodies, interleukin-6 and tumour necrosis factor alpha.^9^

Several studies have shown altered cerebral metabolism in neuropsychiatric lupus. Thus, SPECT scans may be diagnostically useful in patients with normal MRI, especially those with a diffuse cognitive presentation (where it is 75%–93% sensitive).^1 9 13^ SPECT scans may be used as a biomarker for the immediate response to immunotherapy,^9 14^ but do not help in subsequent monitoring or predicting further neuropsychiatric events. 15

Patients with psychiatric symptoms should receive antidepressant and antipsychotic agents.^1^ Electroconvulsive therapy was effective in three cases with severe psychosis.^16^ Patients with cognitive decline often respond to glucocorticoids and other immunosuppressants^1^, although corticosteroids can aggravate the psychiatric symptoms. There is evidence for using cyclophosphamide and plasma exchange (sometimes synchronised) in refractory cases^1 17^ and for rituximab in severe refractory cases.^9 13 14 18^ Patients with either a long disease duration or with more than one of the American College of Rheumatology syndromes (table 1)^13^ have a poorer therapeutic prognosis.^9^ Although several CSF cytokine, chemokine and growth factors have been used as markers of the inflammatory process, a more specific biomarker would greatly improve diagnostic and therapeutic research into this rare condition.^9^

**Table 1:** American College of Rheumatology Neuropsychiatric Systemic Lupus Erythematosus classification (1999)

Central nervous system	Peripheral nervous system
Aseptic meningitis	Acute inflammatory demyelinating polyradiculopathy
Cerebrovascular disease	Autonomic disorder
Demyelinating syndrome	Mononeuropathy (single/multiple)
Headache	Myasthenia gravis
Movement disorder (chorea)*	Cranial neuropathy
Myelopathy	Plexopathy
Seizure disorder*	Polyneuropathy
Acute confusional state*	
Anxiety disorder	
Cognitive dysfunction*	
Mood disorder*	
Psychosis*	

*Presentations associated with diffuse neuropsychiatric lupus.

## References

[1] Bertsias GK, Ioannidis JP, Aringer M, et al EULAR recommendations for the management of systemic lupus erythematosus with neuropsychiatric manifestations: report of a task force of the EULAR standing committee for clinical affairs. Ann Rheum Dis 2010;69:2074–82. 10.1136/ard.2010.130476 20724309

[2] The American College of Rheumatology nomenclature and case definitions for neuropsychiatric lupus syndromes. Arthritis Rheum 1999;42:599–608. 10.1002/1529-0131(199904)42:4<599::AID-ANR2>3.0.CO;2-F 10211873

[3] Joseph FG, Scolding NJ Neurolupus. Pract Neurol 2010;10:4–15. 10.1136/jnnp.2009.200071 20130291

[4] Brey RL, Holliday SL, Saklad AR, et al Neuropsychiatric syndromes in lupus: prevalence using standardized definitions. Neurology 2002;58:1214–20. 10.1212/WNL.58.8.1214 11971089

[5] Arinuma Y, Kikuchi H, Wada T, et al Brain MRI in patients with diffuse psychiatric/neuropsychological syndromes in systemic lupus erythematosus. Lupus Sci Med 2014;1:e000050 10.1136/lupus-2014-000050 25396069 PMC4225739

[6] Postal M, Costallat LTL, Appenzeller S Neuropsychiatric Manifestations in Systemic Lupus Erythematosus. CNS Drugs 2011;25:721–36. 10.2165/11591670-000000000-00000 21870886

[7] Aranow C, Diamond B, Mackay M Glutamate receptor biology and its clinical significance in neuropsychiatric systemic lupus erythematosus. Rheum Dis Clin North Am 2010;36:187–201. 10.1016/j.rdc.2009.12.007 20202599 PMC2837540

[8] Kivity S, Agmon-Levin N, Zandman-Goddard G, et al Neuropsychiatric lupus: a mosaic of clinical presentations. BMC Med 2015;13:43 10.1186/s12916-015-0269-8 25858312 PMC4349748

[9] Ichinose K, Arima K, Umeda M, et al Predictors of clinical outcomes in patients with neuropsychiatric systemic lupus erythematosus. Cytokine 2016;79:31–7. 10.1016/j.cyto.2015.12.010 26745468

[10] Scolding NJ, Joseph FG The neuropathology and pathogenesis of systemic lupus erythematosus. Neuropathol Appl Neurobiol 2002;28:173–89. 10.1046/j.1365-2990.2002.00406.x 12060342

[11] Zardi EM, Taccone A, Marigliano B, et al Neuropsychiatric systemic lupus erythematosus: tools for the diagnosis. Autoimmun Rev 2014;13:831–9. 10.1016/j.autrev.2014.04.002 24704869

[12] Zivadinov R, Shucard JL, Hussein S, et al Multimodal imaging in systemic lupus erythematosus patients with diffuse neuropsychiatric involvement. Lupus 2013;22:675–83. 10.1177/0961203313486193 23640981

[13] Castellino G, Padovan M, Bortoluzzi A, et al Single photon emission computed tomography and magnetic resonance imaging evaluation in SLE patients with and without neuropsychiatric involvement. Rheumatology 2008;47:319–23. 10.1093/rheumatology/kem354 18218648

[14] Narváez J, Ríos-Rodriguez V, de la Fuente D, et al Rituximab therapy in refractory neuropsychiatric lupus: current clinical evidence. Semin Arthritis Rheum 2011;41:364–72. 10.1016/j.semarthrit.2011.06.004 21875742

[15] Castellino G, Bortoluzzi A, Padovan M, et al Repeated brain conventional MRI and SPECT evaluation in systemic lupus erythematosus patients with and without neuropsychiatric involvement: a follow up study. Lupus 2011;20:1387–95. 10.1177/0961203311415304 21946513

[16] Tan LP, Tan LE Electroconvulsive therapy for severe neuropsychiatric lupus with psychosis. J ECT 2013;29:243–6. 10.1097/YCT.0b013e3182809c01 23535497

[17] Neuwelt CM The role of plasmapheresis in the treatment of severe central nervous system neuropsychiatric systemic lupus erythematosus. Ther Apher Dial 2003;7:173–82. 10.1046/j.1526-0968.2003.00032.x 12918940

[18] Cobo-Ibáñez T, Loza-Santamaría E, Pego-Reigosa JM, et al Efficacy and safety of rituximab in the treatment of non-renal systemic lupus erythematosus: a systematic review. Semin Arthritis Rheum 2014;44:175–85. 10.1016/j.semarthrit.2014.04.002 24830791

